# A Rapid Prediction Method for Underwater Vehicle Radiated Noise Based on Feature Selection and Parallel Residual Neural Network

**DOI:** 10.3390/s26010266

**Published:** 2026-01-01

**Authors:** Fang Ji, Ziming Li, Weijia Feng, Mengxi Shi, Xiang Ji

**Affiliations:** China Ship Research and Development Academy, Beijing 100101, China; heuliziming@163.com (Z.L.);

**Keywords:** data-driven, underwater radiated noise prediction, neural network, feature selection

## Abstract

Efficient and high-precision prediction of underwater vehicle radiated noise is crucial for warship stealth assessment. To overcome the high modeling complexity and limited prediction capability of traditional methods, this paper proposes ADE-PNN-ResNet, a fast underwater radiated noise (URN) prediction model integrating Adaptive Differential Evolution (ADE) with a Parallel Residual Neural Network (PNN-ResNet). This data-driven framework replaces conventional physics-based modeling, significantly reducing complexity while preserving high prediction accuracy. This study includes three core points: Firstly, for each 1/3-octave target noise band, a joint feature selection strategy of measurement points and frequency bands based on the ADE is proposed to provide high-quality inputs for the subsequent model. Secondly, a Parallel Neural Network (PNN) is constructed by integrating Radial Basis Function Neural Network (RBFNN) that excels at handling local features and Multi-Layer Perceptron (MLP) that focuses on global features. PNN is then cascaded via residual connections to form PNN-ResNet, deepening the network layers and efficiently capturing the complex nonlinear relationships between vibration and noise. Thirdly, the proposed ADE-PNN-ResNet is validated using vibration and noise data collected from lake experiments of a scaled underwater vehicle model. Under the validation conditions, the absolute prediction error is below 3 dB for 96% of the 1/3-octave bands within the frequency range of 100–2000 Hz, with the inference time for prediction taking merely a few seconds. The research demonstrates that ADE-PNN-ResNet balances prediction accuracy and efficiency, providing a feasible intelligent solution for the rapid prediction of underwater vehicle radiated noise in engineering applications.

## 1. Introduction

With the increasing activity of marine development and the widespread application of various underwater equipment such as unmanned underwater vehicles (UUVs) and offshore platform, the underwater radiated noise (URN) generated by structural vibration has exerted an increasingly significant impact on the marine ecological environment, operational efficiency, and military security. Accurate prediction of the radiated noise under different operating conditions can help assess the exposure risk of underwater vehicles in complex combat scenarios and provide a scientific basis for tactical deployment.

Among traditional prediction methods, analytical methods [1,2,3] are limited to simplified regular shells with typical boundary conditions, while numerical methods [4,5,6,7] suffer from complex modeling procedures and poor generalization ability. Neither the prediction time nor the accuracy of these two methods can satisfy the requirements of practical engineering. To address these limitations, data-driven models have emerged. Based on Automatic Identification System (AIS) data and extensive measured ship noise data, MacGillivray et al. [8] proposed the JOMOPANS-ECHO model—a semi-empirical statistical regression model for predicting underwater ship noise source levels. This model is computationally efficient and achieves a prediction error with a standard deviation of approximately 6 dB across the 20 Hz–20 kHz frequency range in 1/3-octave bands. Building on this work, Lloyd et al. [9] proposed the PIANO model, which integrates physical principles with semi-empirical data. Unlike traditional semi-empirical models that rely solely on basic AIS parameters, PIANO model explicitly distinguishes the physical sources of propeller cavitation noise and machinery noise, incorporates key design parameters, but has a slightly higher prediction uncertainty than conventional models. From the perspective of existing research progress, the transfer function method [10,11,12,13,14] and its derived technologies have become relatively efficient and high-precision strategies in the field of underwater vehicle radiated noise prediction. The Operational Transfer Path Analysis (OTPA) method [15,16] decomposes the contributions of different excitation sources and transfer paths to noise by analyzing a large amount of real data and then uses real-time vibration monitoring data as the input of the transfer function to predict radiated noise. Javier et al. [17] verified the correlation between vibration monitoring data and noise using coherence functions, optimized the sensor layout positions, and ultimately achieved a prediction error of less than 2 dB. Field tests of the German Navy’s OMS underwater noise monitoring and acoustic fingerprint prediction system indicate that when measurement points fail to fully cover critical noise sources, predicted values may be underestimated by approximately 6 dB [18]. The above studies indicate that the selection of measurement point positions has a decisive impact on the OTPA noise prediction accuracy.

Based on vessel type, speed, dimensions, tonnage, and environmental parameters such as wind and current, Johnson et al. [19] used MLP to predict the monopole source level (MSL) of commercial vessels in the Gulf of Mexico, achieving a median error of 3 dB across 1/3 octave bands from 20 to 1000 Hz. However, this method entails high data acquisition costs and, to some extent, neglects the physical mechanisms underlying noise generation and propagation. To address the above problems, recent studies have leveraged data-driven machine techniques to directly learn the complex nonlinear mapping relationship between vibration acceleration measurements at selected points and radiated noise data at target positions [20,21], eliminating the need for prior modeling of transmission paths. Underdetermined equation problem arises when samples are insufficient. Although truncated singular value decomposition and regularization techniques can partially alleviate this issue [22], the model’s sensitivity to specific frequency bands still limits its robustness. Huang et al. [21] constructed an improved Bagging ensemble model with ridge regression as the base learner, and synchronously optimized sensor configuration and frequency band selection using the Differential Evolution (DE) algorithm, achieving noise prediction errors controlled within 3 dB for 97% of the 1/3 octave bands. Park et al. [23] combined neural networks with traditional OTPA to preserve acoustic information and solve underdetermined equation problems. Ye et al. [24] proposed a data augmentation method based on an improved Gaussian mixture model (GMM) to address insufficient coverage in sparsely sampled regions. The enhanced data served as input for a Lasso regression surrogate model, achieving noise prediction errors below 2 dB.

Research on utilizing neural networks to train surrogate models for predicting underwater vehicle noise remains relatively scarce, despite the widespread application of such methods in related fields for modeling input–output relationships and solving regression problems. Commonly used methods for constructing surrogate models primarily include radial basis function neural network (RBFNN) [25,26], multilayer perceptron (MLP) [27,28], backpropagation neural network (BPNN) [29,30], and support vector machine (SVM) [31,32].

Feature selection aims to identify informative features that not only provide high-quality inputs for subsequent models but also reduces feature dimensionality to alleviate ill-posed problems. Based on different mechanisms, existing feature selection methods can be categorized into three major types: filtering, wrapping, and embedding. Among them, wrapper methods prioritize prediction model performance to search for optimal feature subsets, making them the most adaptable choice for underwater vehicle radiated noise prediction scenarios. Intelligent optimization algorithms are widely adopted in feature selection to enhance data quality and improve model performance [33]. Among numerous swarm-inspired heuristic algorithms, Differential Evolution (DE) [34] exhibits distinct advantages due to its robustness and unique search mechanism. Against this background, this paper proposes a Parallel Residual Connection Neural Network (PNN-ResNet), which integrates RBFNN and MLP in a parallel structure. Adaptive differential evolution (ADE) algorithm is integrated at the front end to select input vibration features, ultimately achieving efficient and high-precision URN prediction. Compared with the methods in references [8,9,19], which rely on multi-source heterogeneous data such as AIS information, vessel parameters, and ocean environmental variables, the proposed approach requires only hull vibration monitoring data. This reduces data acquisition costs and enhances engineering deployment ability, making it more suitable for real-time, high-precision noise prediction for individual vessels. Main contributions of this paper are as follows:

(1) Aiming at the problems of high dimensionality in underwater vehicle vibration data, as well as model overfitting and poor generalization ability caused by redundant features that weaken data correlation, a feature selection method based on the ADE is proposed. By extracting the optimal feature subset, this method effectively reduces data dimensionality, enhances feature representativeness, and provides high-quality inputs for the subsequent model.
(2) PNN-ResNet combining RBFNN and MLP in parallel is proposed to model the transfer function between underwater vehicle vibration and radiated noise. The RBFNN captures local nonlinear correlations, while the MLP extracts global complex patterns through feature abstraction. These two networks form a parallel module PNN, which is then stacked in series via residual connections. This design not only enhances the modeling of deep nonlinear mapping relationships but also avoids gradient vanishing, realizing the collaborative complementarity of local and global features.
(3) The proposed prediction method is validated using vibration and noise data collected from lake experiments of a scaled underwater vehicle model. Under four randomly selected validation conditions, the PNN-ResNet model achieves absolute errors below 2 dB in 70% of the 56 one-third-octave bands (100–2000 Hz), and below 3 dB in 91% of them. By integrating ADE at the PNN-ResNet front-end to screen features of the prediction bands, ADE-PNN-ResNet model achieves a notable performance boost: 91% of the bands achieve an absolute error below 2 dB and 96% below 3 dB. This model provides a feasible engineering implementation approach for real-time monitoring of underwater vehicle radiated noise in actual marine environments.

## 2. Model Construction

### 2.1. Underwater Vehicle Radiated Noise Prediction Model

Linear machine learning algorithms construct predictive model through linearly weighted combinations of input features and typically rely on ensemble learning strategies to capture nonlinear relationships within the data. In contrast, neural networks inherently possess nonlinear modeling capabilities. Deep neural networks can progressively transform raw features into highly abstract nonlinear feature representations by stacking multiple layers of nonlinear transformations, thereby directly establishing complex nonlinear correlations between inputs and outputs.

To comply with acoustic measurement standards and facilitate engineering implementation, this paper adopts the 1/3-octave band vibration data from each measuring point of the underwater vehicle as the model input. This strategy not only preserves essential noise characteristics, but also significantly reduces computational cost. Noise in different frequency bands correlates with specific measurement points and vibration frequency bands. Therefore, this paper introduces multi-band vibration features for modeling. However, this leads to a multiplicative increase in feature dimensionality as the number of frequency bands grows. To address this, feature selection is employed to reduce input dimensionality, eliminate redundant features, and alleviate the underdetermined problem to ensure the model maintains stable and reliable prediction performance even under small-sample scenarios.

**Figure 1:**
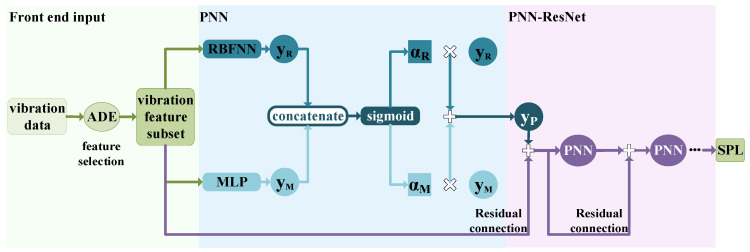
Underwater vehicle radiated noise prediction model.

The prediction model primarily comprises two major components: feature selection—ADE and neural network—PNN-ResNet. The feature selection module identifies vibration features that significantly contribute to noise in a specific frequency band, including measurement points and frequency bands; PNN-ResNet models the nonlinear relationship between these selected vibration features and corresponding radiated noise. The complete underwater vehicle radiated noise prediction model is illustrated in Figure 1. By integrating the trained models for all individual frequency bands, the framework enables rapid full-band noise prediction of underwater vehicles based on real-time vibration monitoring data. Specific design and implementation details are elaborated in Section 2.2 and Section 2.3.

### 2.2. Adaptive Differential Evolution (ADE) Feature Selection Module

Differential Evolution (DE) [34] was proposed by Rainer Stom and Kenneth Price in 1995 to solve Chebyshev polynomials. Inspired by evolutionary concepts like genetic algorithms, DE simulates mutation, crossover, and selection operations within a population to continuously evolve candidate solutions toward the global optimum. It is a multi-objective evolutionary algorithm (MOEA) for continuous variable optimization. Unlike Genetic Algorithm (GA), which rely on random crossover and mutation, or Particle Swarm Optimization (PSO), which depends on individual and collective experience, the mutation operation of DE generates new candidate solutions through directed perturbation based on vector differences, combined with probabilistic crossover operations to iteratively update solutions. This mechanism utilizing the population’s own distribution information (vector differences) enables it to adjust the search step size more intelligently. Consequently, DE achieves an outstanding balance and stability between exploration (searching unexplored regions) and exploitation (refining high-quality regions), offering significant advantages in convergence stability and speed. Additionally, DE requires relatively few manually tuned parameters, and its straightforward crossover and mutation computations result in short iteration times.

**Figure 2:**
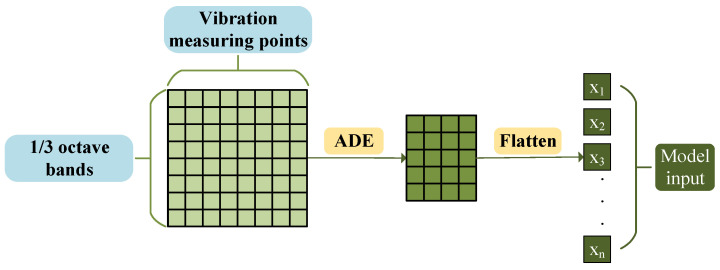
Feature selection flowchart.

Based on the aforementioned advantages, this paper uses Adaptive Differential Evolution (ADE) to select the vibration feature subset with strong correlation to the target frequency band noise, and the fitness value is defined as the prediction error during neural network training. The specific process is illustrated in Figure 2: each row of the original input data represents the acceleration level of each structural vibration measurement point under a specific frequency band. The ADE algorithm implements a two-dimensional screening of vibration measurement points and 1/3 octave bands, and the resulting highly correlated features are flattened into a single row as the fully connected input for the subsequent neural network.

ADE Algorithm Flow:

I. Population Initialization: Randomly generate *NP* individuals in the solution space. Each individual has a dimension equal to the number of candidate features, which comprises measurement points *p* and frequency bands *q*. An individual is represented as:

(1) $$\begin{array}{c} X_{i}(0)=(A_{i,1}(0),A_{i,2}(0),\cdots ,A_{i,p}(0),\cdots ,B_{i,1}(0),B_{i,2}(0),\cdots ,B_{i,q}(0))\\ i=1,2,3,\cdots ,NP \end{array}$$

where the *i*-th individual in the 0-th generation population $X_{i}(0)$ is encoded such that each of its elements follows a uniform distribution over the solution space [0, 1]. This continuous encoding establishes a mapping relationship between the continuous search process and the discrete decision outcomes.

II. Mutation: Vector differences between individuals in the population are used as perturbation. These differences are scaled and then subjected to vector operations with the target individual for mutation, thereby generating a directional mutation vector by leveraging the inherent distribution information of population. The mutation vector for generation *g* + 1 is derived from individuals of generation *g* via Equation (2):

(2) $$V_{i}(g+1)=X_{best}(g)+F(X_{r1}(g)-X_{r2}(g)+X_{r3}(g)-X_{r4}(g))$$

where $X_{best}(g)$ is the optimal individual in the *g*-th generation; $X_{r1}(g)$, $X_{r2}(g)$, $X_{r3}(g)$, $X_{r4}(g)$ are three distinct individuals randomly selected from the current population; the scaling factor *F* is usually set within the range [0, 2]. An excessively large *F* leads to late-stage oscillation and non-convergence, while an excessively small *F* tends to cause premature convergence. The adaptive *F* algorithm dynamically adjusts the scaling factor according to the search state during operation. In the early stage of search, when the population is dispersed and extensive exploration is required, a larger *F* value encourages the algorithm to perform large-step exploration and quickly locate promising regions. In the late search stage, when the population converges near the optimal solution and fine-grained search is required, reducing *F* enables the algorithm to exploit within a small range and stably converge to high-quality solutions. The adaptive scaling factor is calculated as:

(3) $$\begin{array}{l} F=F_{0}\cdot 2^{\lambda }\\ \lambda =e^{1-\frac{Gm}{1+G_{m}-G}} \end{array}$$

where *G_m_* is the total number of iterations set, and *G* is the current iteration count. Individuals after mutation must still satisfy the solution’s boundary conditions.

III. Crossover: This operation constitutes the core mechanism of the ADE. New individuals are generated by performing crossover between the *g*-th generation population individual $X_{i}(g)$ and the mutation vector $V_{i}(g+1)$. Each component of the crossover individual is generated using Equation (4), achieving a balance between global search and local optimization:

(4) $$u_{i,j}(g+1)=\left\{\begin{array}{ll} v_{i,j}\left(g+1\right) & ,\;r_{i}\leq CR\;\text{or rand\_index}\\ x_{i,j}(g) & ,\;\mathrm{else} \end{array}\right.$$

where *CR* is the crossover probability, which controls the probability of retaining genes from the mutation vector; $r_{i}$ is a random number between 0 and 1 generated independently for each component.

IV. Selection: ADE selects individuals for the next generation based on fitness values calculated using the greedy algorithm applied to the current population.

(5) $$X_{i}(g+1)=\left\{\begin{array}{ll} U_{i}(g+1), & \mathrm{if}\;f(U_{i}(g+1))\leq f(X_{i}(g))\\ X_{i}(g), & \mathrm{otherwise} \end{array}\right.$$

**Figure 3:**
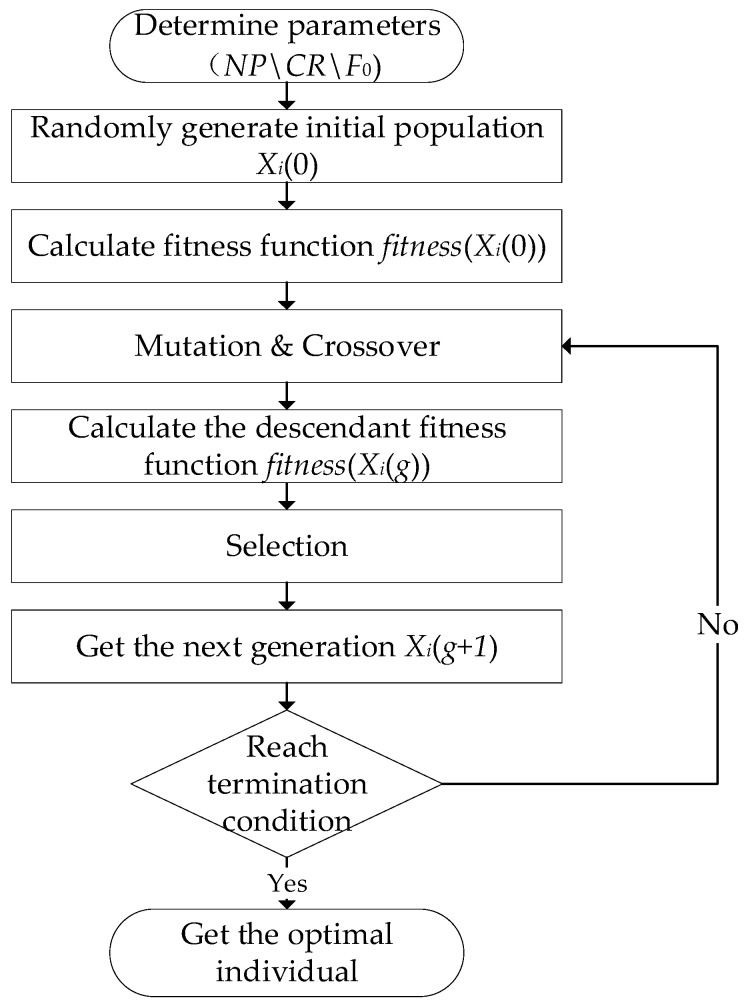
ADE algorithm flowchart.

Repeat steps II–IV until the termination criteria are met. The ADE algorithm flowchart is shown in Figure 3.

The population update is stopped if any of the following termination conditions are met: 1. The specified number of iterations is reached; 2. The fitness value changes slightly over a certain number of iterations; 3. The diversity of the population is poor.

After decoding the optimal individual from the population, the optimized feature index can be obtained:

(6) $$\begin{array}{l} P=where(A_{i,p}>0.5)\\ Q=where(B_{i,q}>0.5) \end{array}$$

where *P* and *Q* represent the vibration measurement point and frequency band index after feature selection, respectively.

The fitness function evaluates the quality of each individual. It is defined as a weighted combination of the root mean square error (RMSE) on the training and validation datasets, balancing the learning ability and generalization capability. Based on this metric, ADE selects the feature subset with the most stable generalization performance. The fitness function formula is as follows:

(7) $$fitness(X_{i})=\alpha \cdot {\mathrm{RMSE}}_{train}(X(P,Q))+(1-\alpha )\cdot {\mathrm{RMSE}}_{val}(X(P,Q))$$

where α is the training dataset weight, X(P,Q) represents the selected features, ${\mathrm{RMSE}}_{train}(X(P,Q))$ and ${\mathrm{RMSE}}_{val}(X(P,Q))$ are the RMSE of the training dataset and validation dataset for the feature selection after training neural network.

### 2.3. Parallel Residual Connection Neural Network PNN-ResNet

The vibration-noise mapping relationship of underwater radiated noise exhibits local sensitivity within specific frequency bands while maintaining global correlations across the entire frequency range. Traditional single-network architectures struggle to capture these features simultaneously. Therefore, this paper designs a Parallel Residual Connection Neural Network (PNN-ResNet) to achieve efficient modeling by integrating complementary local-global features and enhance nonlinear expressions via residual.

#### 2.3.1. A Neural Network with Parallel Architecture of RBFNN and MLP (PNN)

**Figure 4:**
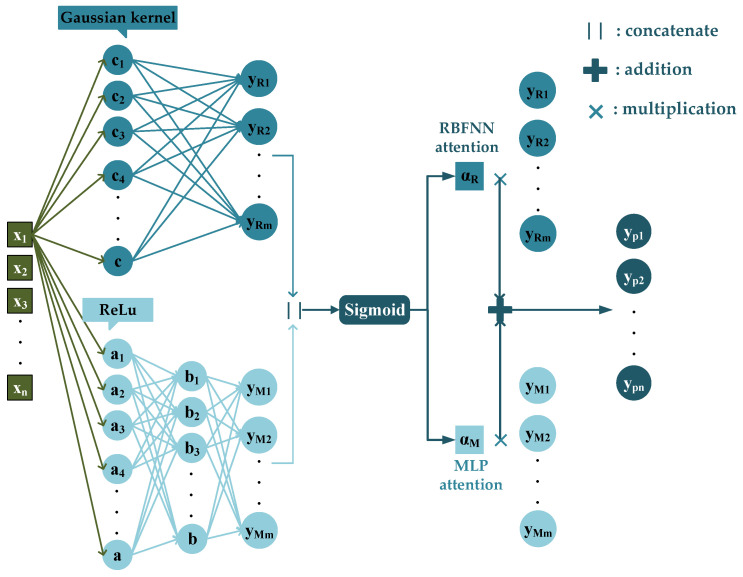
PNN specific structure diagram.

The selected features are fed into the neural network model and fully connected to the hidden layer. The RBFNN maps input data to a high-dimensional space and extracts nonlinear features via multi-scale Gaussian kernels to simultaneously capture local features at different levels. The MLP learns global feature correlations through a multi-layer fully connected network, forming feature complementarity with the RBFNN pathway. The sigmoid function is used to dynamically adjust the output weights of the RBF and MLP pathways, enhancing key features and suppressing noise. The specific architecture of PNN is shown in Figure 4.

RBFNN is a three-layer feedforward neural network whose core principle is to treat input data as a discrete point set to be approximated by constructing a network structure that can approximate the target function using the activation functions of hidden layer nodes. RBFNN consists of an input layer, a hidden layer, and an output layer. The input layer simply transmits input data to the hidden layer without any data processing. The hidden layer is the core component of the network. The neuron uses radial basis function as the activation function to map input data into a high-dimensional space. This transformation converts linearly inseparable problems in low-dimensional space into linearly separable ones in high-dimensional space, thereby enabling effective modeling of nonlinear problems. In this paper, the Gaussian function is selected as the activation function for the RBFNN hidden layer:

(8) $$\phi_{i}\left(x\right)=\exp\left(-\frac{{\left\|x-c_{i}\right\|}^{2}}{2\sigma_{i}^{2}}\right)$$

where *x* denotes input vector containing *n* features; $c_{i}$ is the *n*-dimensional sample center of the *i*-th neuron, which is initialized using the *K*-means clustering algorithm in this paper; $\left\|x-c_{i}\right\|$ represents the Euclidean distance between the input vector and the center point; $\sigma_{i}$ controls the scale of the *i*-th basis function and determines the local response range of the function. The local approximation ability of RBFNN fundamentally stems from the selective activation mechanism of basis function scale control: a basis function is activated only when the input vector falls in the local region around its center. This activation mechanism enables the network to dynamically adjust merely a small subset of associated weights to constrain the optimization search space. This not only accelerates convergence but also reduces the risk of overfitting and entrapment in local minima. In the context of underwater radiated noise prediction, the local approximation advantage of RBFNN provides crucial support for efficiently modeling the frequency-band-specific local abrupt variations in the vibration-noise mapping relationship. The final output of the RBFNN is:

(9) $$y=W_{proj}^{T}\phi_{i}(x)$$

**Figure 5:**
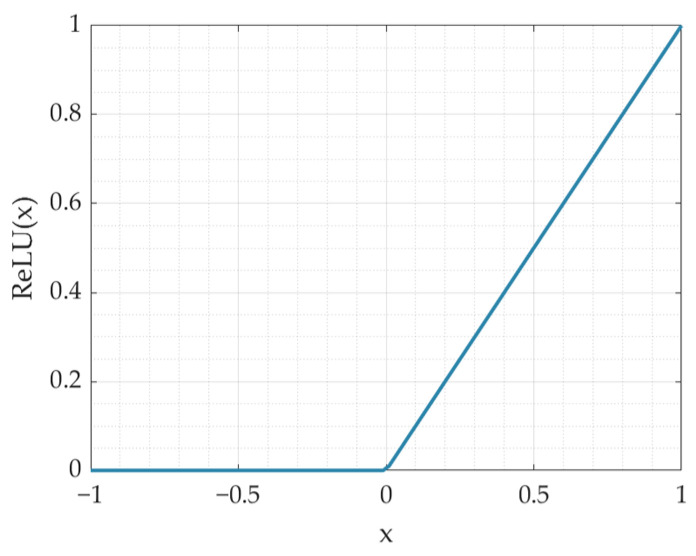
ReLU function curve diagram.

RBFNN can effectively capture local features in nonlinear function approximation. To complement this capability with global feature modeling, this paper integrates the RBFNN in parallel with MLP, also known as artificial neural network (ANN). The MLP typically consists of an input layer, one or more hidden layers, and an output layer. Layers are linearly fully connected, and activation functions are used between them to introduce nonlinear and extract global features of the input. In this paper, the Rectified Linear Unit (ReLU) shown in Figure 5 is selected as the activation function to avoid gradient vanishing during training. Additionally, dropout layers are used in MLP to randomly deactivate a fraction of neurons, training a different “sub-network” each iteration. This prevents over-reliance on specific neurons, thereby reducing co-adaptation and improving model robustness and generalization.

(10) ReLU(x)=max(0, x)

The input-output relationship of hidden layer neurons is:

(11) $$y_{j}=\mathrm{ReLU}(\sum_{i=1}^{n}\omega_{ji}x_{i}+b_{j})$$

where $x_{i}$ is the output of previous layer neurons; $\omega_{ji}$ is the connection weight for the *j*-th neuron; and $b_{j}$ is the bias term.

**Figure 6:**
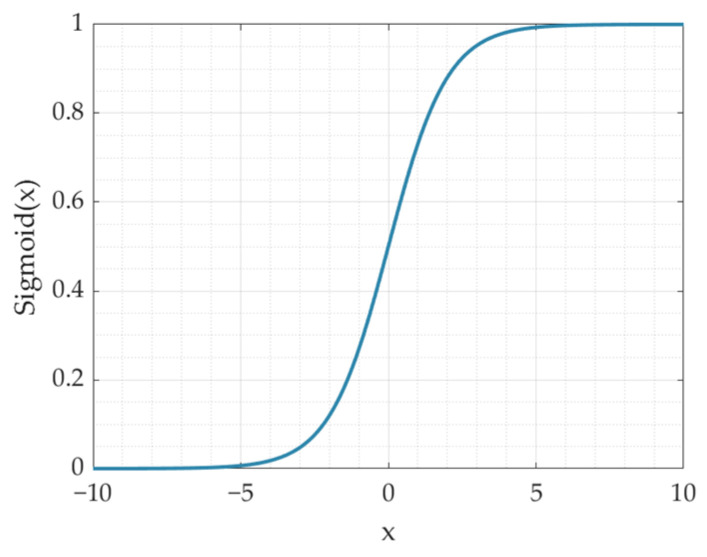
Sigmoid function curve diagram.

To achieve adaptive balance of local and global features, this paper adopts the sigmoid attention mechanism to fuse the outputs of the RBFNN and MLP pathways. First, the outputs of both pathways are directly concatenated to obtain the feature $C=[y_{R}||y_{M}]$, which is then fed into a fully connected layer for linear transformation. After processing by the sigmoid activation function shown in Figure 6, attention weights for the two pathways are obtained, which are mapped to the range [0, 1] with independent weights for each pathway:

(12) $$\mathrm{sigmoid}(x)=\frac{1}{1+e^{-x}}$$

(13) $$\alpha =\mathrm{sigmoid}(\omega_{f}\cdot C+b_{f})$$

(14) $$\alpha_{R}=\alpha [1],\;\alpha_{M}=\alpha [2]$$

where α is a 2D vector corresponding to the weights of the RBFNN $\alpha_{R}$ and MLP $\alpha_{M}$.

Finally, the obtained weights are multiplied by the outputs of the corresponding pathways, respectively, to achieve feature fusion. This mechanism dynamically allocates weights through data-driven learning.

(15) $$y_{p}=\alpha_{R}\cdot y_{R}+\alpha_{M}\cdot y_{M}$$

#### 2.3.2. Residual Connection

**Figure 7:**
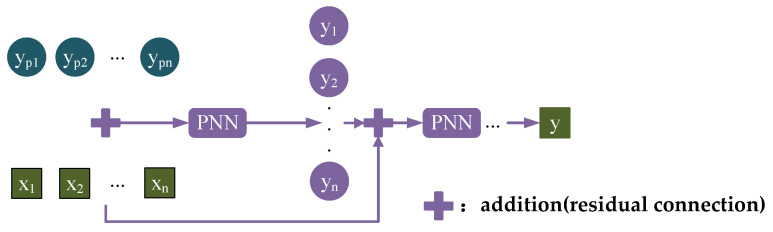
Residual connection diagram.

To further enhance nonlinear representation ability, this paper proposes a multi-stage cascaded PNN architecture via residual connection—PNN-ResNet. As network depth increases, the model may encounter gradient vanishing problem, leading to unstable training and optimization difficulties. To mitigate this issue, a residual connection mechanism is introduced to directly propagate features from previous stages to subsequent layers. The input features selected by ADE are added to the output of the first parallel neural network PNN module to serve as the input for the next parallel module. This process is repeated sequentially. By enabling direct information flow through skip connections, the residual structure alleviates gradient decay in deep networks, stabilizes training, and enables feature reuse, further enhancing expression efficiency and generalization performance. The final output is the noise value for the target frequency band, as shown in Figure 7.

## 3. Data Acquisition and Preprocessing

### 3.1. Experimental Conditions

**Figure 8:**
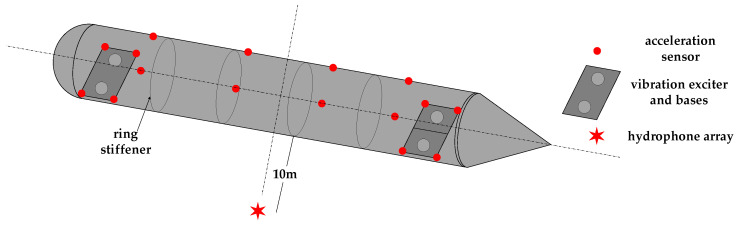
Schematic diagram of scaled model structure and instrument layout.

**Figure 9:**
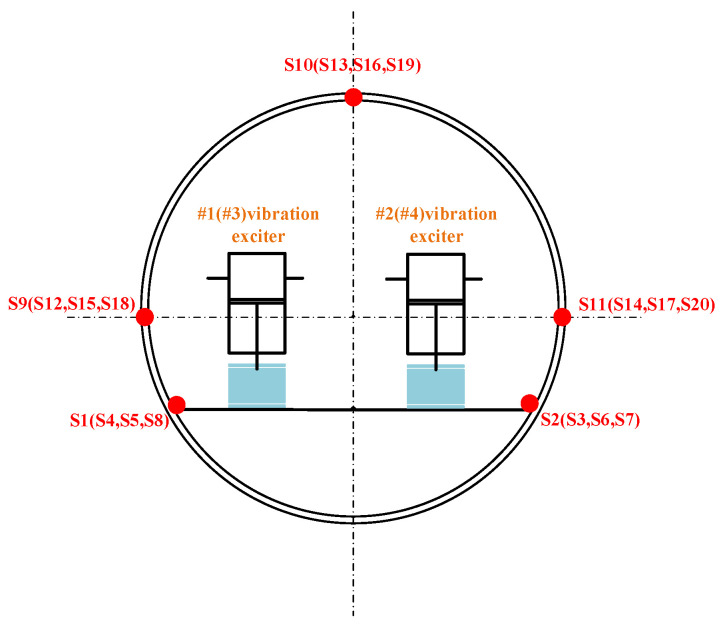
Schematic diagram of instrument layout at the bow cross-section.

**Figure 10:**
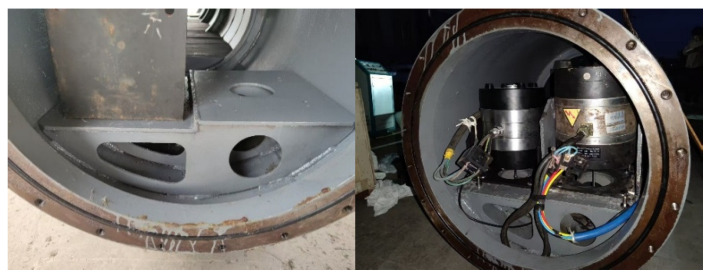
Physical diagram of the base and vibration exciters.

To evaluate the performance of the proposed model, a vibration-acoustic measurement experiment was conducted on a self-built scaled model in Qiandao Lake, Hangzhou. As shown in Figure 8, the model has a three-segment structure (forward spherical shell, mid-section stiffened cylindrical shell, aft conical shell) with key dimensions: overall length 6.4 m, diameter 0.7 m, and ring stiffener spacing 0.9 m. Vibration exciters (#1–#4) were installed on two bases (bow: #1–#2; stern: #3–#4). Acceleration sensors (S1–S8) were placed at four corners of the two bases. Four groups of acceleration sensors (3 sensors per group, S9–S20) were uniformly arranged on the inner wall of the cylindrical shell. For each group, one sensor was at the top of the model, and the other two were at the midpoints of the port and starboard sides, with an interval of 1.5 m between adjacent groups. Detailed layouts are shown in Figure 8 and Figure 9. The layout of the base and vibration exciters at the test site is shown in the Figure 10. An 8-element vertical hydrophone array was deployed 10 m away from the midship section of the model, with the hydrophones positioned at depths of 2 m, 4 m, 5 m, 6 m, 8 m, 10 m, 12 m, and 14 m below the water surface.

To approximate the actual operating conditions of underwater vehicles and ensure the diversity and reliability of the dataset, the experiment generated six sets of excitation states by controlling the vibration exciters. Each set includes three types of excitation responses to simulate different speeds (low, medium, and high) and different model submergence depths (5 m, 10 m, and 13 m). Detailed excitation states are presented in Table 1, where “on” indicates the vibration exciter is activated and “-” indicates it is deactivated. A total of 54 sets of time-domain signals from vibration acceleration sensors and hydrophones were synchronously collected, with a sampling rate $f_{s}$= 25,000 Hz and an acquisition duration of 20 s, which is consistent with the vibration period of the exciters.

### 3.2. Data Processing Methods

To match the input format of the prediction model, the experimental data need be processed as follows. First, convert the collected time-domain signals of vibration and noise to the frequency domain using Fourier transform, and calculate the bilateral spectral amplitude at $f_{k}=k\frac{f_{s}}{N}$.

(16) $$\begin{array}{l} X[k]=\sum_{n=0}^{N-1}x[n]\cdot e^{-j2\pi kn/N}\\ A[k]=\frac{2}{N}\left|X[k]\right| \end{array}$$

The sound pressure spectrum level $L_{p}$ and vibration acceleration spectrum level $L_{v}$ are given by

(17) $$\begin{array}{l} L_{p}=20\mathrm{lg}\frac{A_{p}[k]}{\sqrt{2}}-20\mathrm{lg}S-20\mathrm{lg}p_{ref}\\ L_{v}=20\mathrm{lg}\frac{A_{v}[k]}{\sqrt{2}}-20\mathrm{lg}v_{ref} \end{array}$$

where *S* is the hydrophone sensitivity, $p_{ref}$ and $v_{ref}$ represent the reference sound pressure and vibration acceleration.

The total band level is calculated by superimposing energies. Taking sound pressure as an example, the 1/3-octave sound pressure level is computed as Equation (18). The frequency range is defined by lower limit $f_{l}=f_{c}\cdot 2^{-1/6}$, upper limit $f_{h}=f_{c}\cdot 2^{1/6}$, and $f_{c}$ is center frequency.

(18) $$SPL(f_{c})=10\mathrm{lg}\left(\sum_{k=f_{l}}^{k=f_{h}}10^{L_{p}/10}\right)$$

## 4. Results Analysis

### 4.1. Dataset Composition and Model Training

The time-domain signals of vibration acceleration and hydrophone noise were processed using the data processing method described in Section 3.2. The received signals from two hydrophones with submersion depths close to that of the model were averaged to obtain the corresponding underwater radiated noise (URN) data. Finally, vibration-noise data of 30 conditions combining different excitation states, ship speeds, and model submergence depths were selected to form the training dataset. Four conditions were randomly chosen as the validation dataset, aiming to verify the prediction accuracy and generalization ability of the model under small training sample scenarios. The specific simulated states are presented in Table 2.

**Figure 11:**
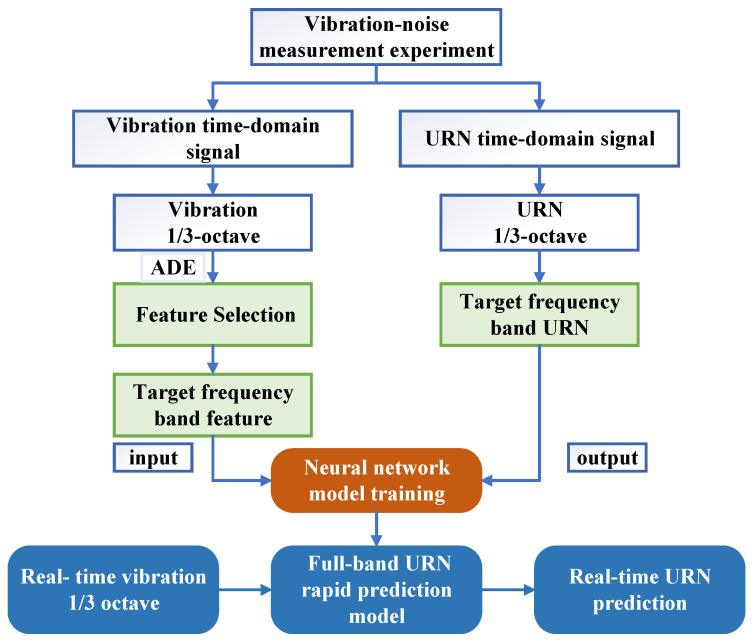
Flow chart of the rapid prediction model for underwater vehicle radiated noise.

The model input is formatted as an Excel spreadsheet. Each row of the vibration data samples corresponds to the 1/3-octave band acceleration levels of all measurement points for a specific frequency band. The associated noise target is provided as a single column of 1/3-octave band sound pressure levels (SPLs), spanning 14 frequency bands from 100 Hz to 2000 Hz. The training workflow of the rapid prediction model for underwater vehicle radiated noise proposed in this paper is illustrated in Figure 11.

The model proposed in this paper is constructed using Python 3.9 programming language and the TensorFlow-GPU 2.7.0 framework. The specific software environment consists of a Windows 11 operating system, Python = 3.9, and TensorFlow-GPU 2.7.0. The hardware configuration includes an NVIDIA GeForce RTX 3060 Laptop GPU and a 12th Gen Intel^®^ Core^TM^ i9-12900H CPU.

To eliminate dimensional discrepancies, the vibration and noise data are normalized separately. RMSE of the training set is adopted as the loss function, and the Adam optimizer was selected with the following configurations: a learning rate of 0.001, an exponential decay rate of 0.9 for the first-order moment estimation, and 0.999 for the second-order moment estimation. Early stopping mechanism was used to monitor the training loss. If the training result showed no improvement after a certain number of epochs, the training process was terminated, and the model with the optimal performance was saved.

### 4.2. Prediction Results of the Neural Network Model

#### 4.2.1. Prediction Results of PNN-ResNet

**Figure 12:**
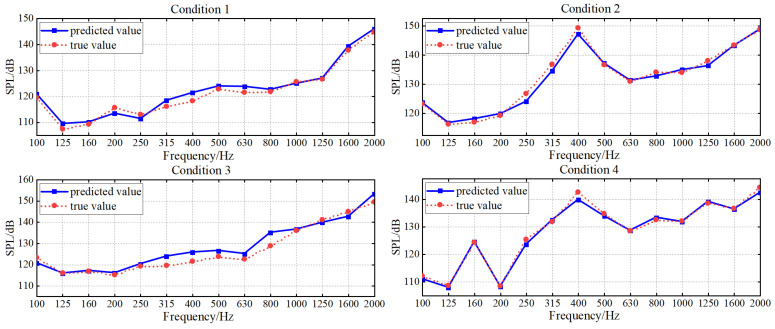
PNN-ResNet prediction results in validation conditions.

**Figure 13:**
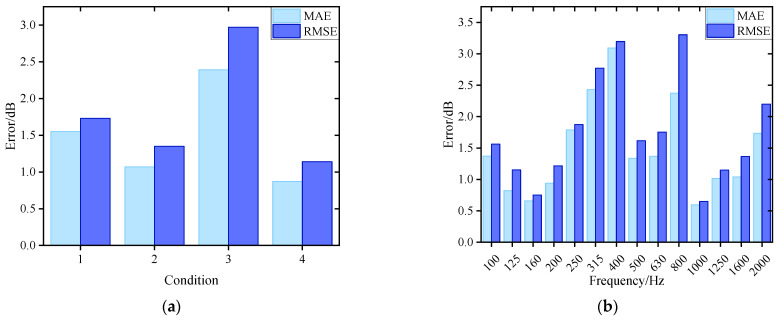
Noise prediction error for PNN-ResNet: (**a**) Prediction error in different conditions; (**b**) Prediction error in 1/3-octave bands.

To accurately model the nonlinear relationship between structural vibration and radiated noise of underwater vehicles, this paper develops a parallel neural network (PNN) composed of RBFNN and MLP to integrate the local approximation capability and the global mapping ability. Residual structure is used to connect the PNN, forming PNN-ResNet to enhance nonlinear expression capability. Grid search and cross-validation are used to tune the hyperparameter of the PNN-ResNet model. The specific settings are as follows: the candidate values for the number of hidden layer neurons in RBFNN are set as {10, 15, 20, 25}. Considering that residual modules increase network complexity and given the heightened risk of overfitting under limited training data, the MLP hidden layer is fixed to two fully connected layers with 64 and 32 neurons, and the number of residual modules is designated as a candidate range of 2 to 10. The optimal configuration is finally determined as 20 hidden layer neurons in RBFNN and 5 residual modules, achieving an optimal balance between fitting accuracy and generalization capability. The noise curves of the true and predicted values for each 1/3-octave band across four validation conditions are shown in Figure 12, and the error values are shown in Figure 13 where (a) targets different validation conditions to reflect global stability, while (b) targets 1/3-octave bands to comprehensively verify the comprehensive performance of the model.

The prediction results of validation dataset demonstrate that the PNN-ResNet model can effectively capture the noise response characteristics under different conditions. The noise curves of Condition 1 and Condition 3 show an overall upward trend with frequency, while peaks appear at 400 Hz in Condition 2 and at 160 Hz and 400 Hz in Condition 4. The prediction results align with these variation trends, and consistently close to the true values. As shown in Figure 13a, the MAE for all validation conditions remains below 2.5 dB. Condition 3 exhibits the poorest overall prediction performance with an MAE of 2.39 dB, while condition 4 achieves the lowest MAE at 0.87 dB. The RMSE for all conditions stays below 3 dB. To comprehensively evaluate the model performance, this study statistically analyzes the prediction errors of all frequency bands for 4 validation conditions, as shown in Figure 13b. The MAE at 315 Hz, 400 Hz, and 800 Hz exceed 2 dB, with the highest MAE of 3.09 dB in the 400 Hz band. For the 800 Hz band, the MAE is 2.37 dB, while its RMSE reaches 3.3 dB, the highest among all frequency bands. As shown in Figure 12, compared with the prediction performance at 800 Hz, the prediction deviation under Condition 3 is significantly larger than that under other conditions, which is an extreme value. This explains why the ratio of MAE to RMSE at 800 Hz is relatively high.

Average error alone cannot fully capture prediction performance across frequency bands or support refined evaluation. To address this limitation, this study further analyzes prediction values of 56 frequency bands (14 1/3-octave bands under 4 validation conditions). Statistical results show that 70% of the 1/3 octave bands achieve an absolute error below 2 dB, and 91% fall within 3 dB. This demonstrates that the model achieves favorable accuracy both globally and in individual frequency bands. Although individual 1/3 octave bands (e.g., 400 Hz, 800 Hz) exhibit error fluctuations affected by outliers, the model still demonstrates strong fitting capability that balances local details and global patterns.

**Figure 14:**
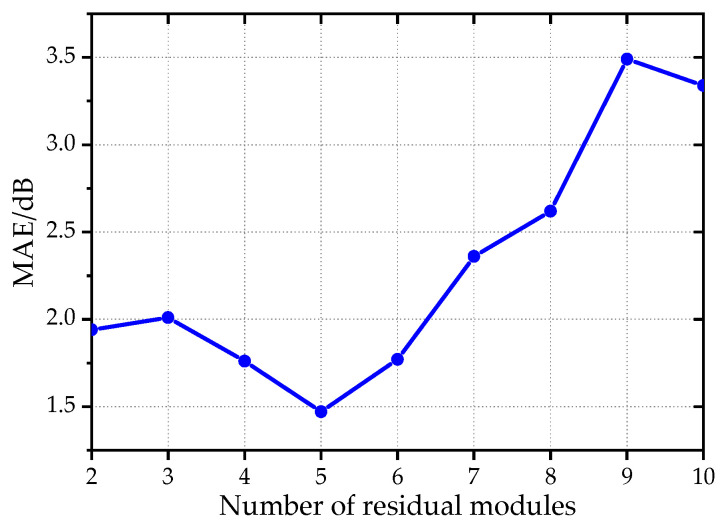
The trend of validation dataset MAE changing with the number of residual modules.

Network complexity has a critical impact on generalization performance under small-sample conditions. To clarify its effect on the PNN-ResNet model, a series of experiments was conducted in which the number of residual modules varied while all other hyperparameters were fixed at their optimal values. The results are summarized in Figure 14. The number of residual modules has a significant effect on the prediction accuracy of the model. When the number of residual modules increases to 5, the MAE of the validation set shows an obvious downward trend, decreasing from 1.94 dB to 1.47 dB. However, further increasing the number of modules, the MAE begins to rise. An excessive number of residual modules leads to redundant network parameters, resulting in overfitting under small-sample training scenarios and thus compromising the generalization performance of the model.

Considering both model accuracy and training efficiency, a rational design of the number of residual modules not only enhances nonlinear expression capability but also avoids overfitting and computational costs increasing, thereby achieving an optimal balance between network depth and model performance.

#### 4.2.2. Ablation Experiment

To verify the effectiveness of the proposed PNN-ResNet model, an ablation study is conducted on its key architectural components and compared with support vector regression (SVR), a classic machine learning model. To ensure a fair and scientific comparison, the hyperparameters of all models are determined via grid search and cross-validation. Specifically, the detailed hyperparameter settings and training schemes (including the Adam optimizer and early stopping mechanism) for the baseline models are consistent with those specified in Section 4.2.1. The prediction errors of different models under four conditions are presented in Table 3, and the model frequency band prediction accuracy is presented in Table 4.

The SVR model exhibits relatively stable performance across all working conditions, with its overall prediction accuracy being superior to that of the MLP and RBFNN, but still inferior to the PNN. MLP and RBFNN exhibit significant differences in prediction accuracy under the four conditions and demonstrate poor generalization capabilities. By fusing these two models, PNN achieves further improvement in overall prediction performance. Although the errors under Conditions 1 and 2 are slightly higher than those of MLP, the PNN significantly reduces the average prediction error and delivers substantial accuracy gains for Conditions 3 and 4 which originally show large prediction deviations. This confirms the effectiveness of the PNN’s hybrid design, which leverages the global fitting capacity of the MLP and the local approximation strength of the RBFNN, thereby demonstrating strong feasibility and robustness. With the incorporation of residual connections, the proposed PNN-ResNet model demonstrates stable performance under all validation conditions, with the average error reduced by 20% compared to the basic PNN. Residual structure further enhances the robustness and generalization ability, fully validating the rationality of the proposed model design.

### 4.3. ADE-PNN-ResNet Prediction Results

Referring to empirical parameter settings in relevant studies on DE [34], the core parameters of ADE are configured as follows: population size (*NP*) is set to 120, ensuring comprehensive coverage of the search space for two-dimensional features; crossover probability (*CR*) is set to 0.85, balancing global exploration while preserving effective genes from mutation vectors; initial scaling factor (*F*_0_) is set to 0.5, preventing premature convergence in the early stages and allowing for fine-tuned adjustments during later phases.

**Figure 15:**
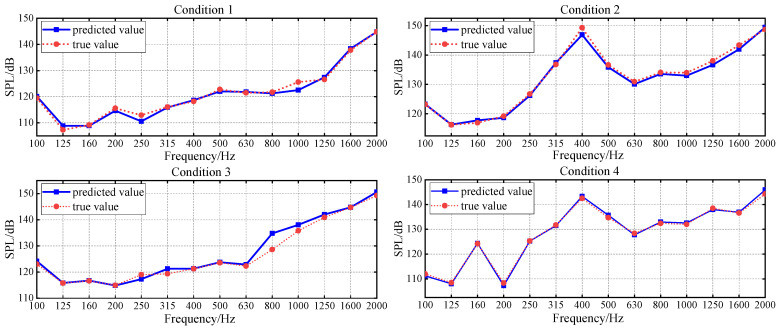
ADE-PNN-ResNet prediction results in validation conditions.

The ADE feature selection module is integrated at the front end of the PNN-ResNet. The results of limited training are used as indicators to search for the optimal vibration feature subset corresponding to each noise frequency band. Given the small size of the validation set, this study sets the weight *α* in Equation (7) to 0.7 to mitigate optimistic bias. After training individual models for each frequency band, they are integrated to construct the overall underwater vehicle radiated noise prediction model. The prediction results of ADE-PNN-ResNet are shown in Figure 15.

**Figure 16:**
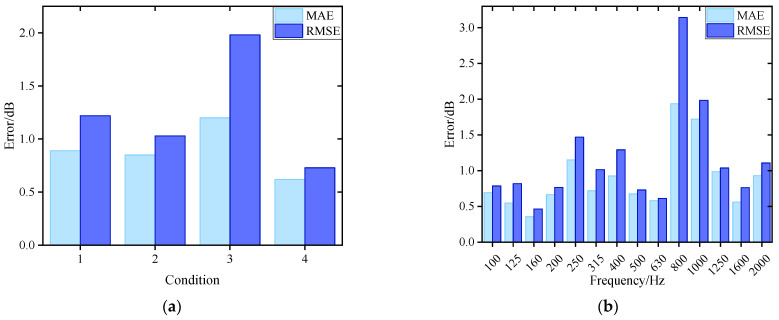
Noise prediction error for ADE-PNN-ResNet: (**a**) Prediction error in different conditions; (**b**) Prediction error in 1/3-octave bands.

**Figure 17:**
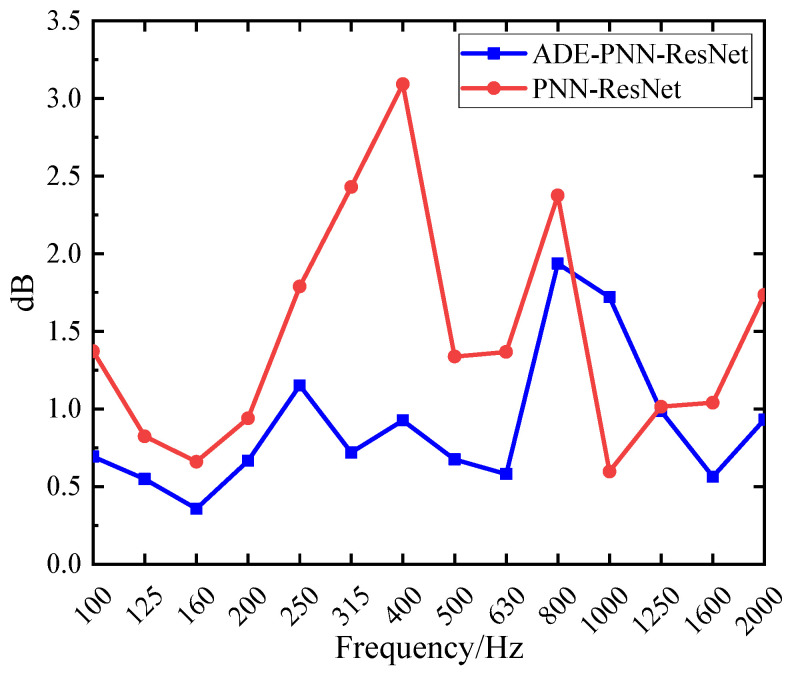
Comparison of prediction MAE among different models.

Compared with the results in Section 4.2.1, the integration of the ADE feature selection module has led to a significant reduction in both the MAE and RMSE across all four validation conditions. As shown in Figure 16a, both metrics are now consistently below 2 dB. Figure 17 presents a comparison of prediction errors before and after the integration of the ADE feature selection module. The PNN-ResNet model exhibited relatively large prediction errors at the 315 Hz, 400 Hz, and 800 Hz frequency bands. After introducing the ADE, the errors at the first two frequency bands were significantly reduced: the MAE at 315 Hz decreased from 2.43 dB to 0.72 dB, and that at 400 Hz dropped from 3.09 dB to 0.93 dB. This demonstrates that feature selection provides the model with higher-quality input, substantially lowering prediction errors and enhancing its generalization capability.

Overall, among the absolute prediction errors across the 56 frequency bands (14 bands × 4 validation conditions), 91% are below 2 dB and 96% are below 3 dB. Taking the 400 Hz band, which demonstrated particularly effective accuracy improvement, as an example. After the synchronous optimization of vibration measurement points and 1/3 octave bands, the dimension of the input feature vector is reduced from 20 × 14 (corresponding to 14 1/3 octave band data from 20 vibration measurement points) to 10 × 4, effectively eliminating redundant measurement points and frequency bands. Table 5 presents the specific two-dimensional features of the selected measurement points and frequency bands. Beyond the primary 400 Hz band, the selected bands include 200 Hz, 630 Hz, and 1250 Hz. It can be observed that the vibration measurement points arranged around the base exhibit a high correlation with noise. The introduction of 1/3 octave bands with stronger relevance further refines the input feature, thereby enhancing prediction accuracy.

However, feature selection requires iterative training to identify the optimal feature subset, which undoubtedly significantly increases the training time of the full-frequency-band prediction model. The computational time is statistically recorded via multiple runs with the network hyperparameters fixed. Even with GPU-accelerated parallel training for multiple frequency bands, the training time for the full-band model with feature selection increases to approximately 20 h, compared to only about 15 min for the model without feature selection under identical computational conditions. Nevertheless, once the model is fully trained, the predicted noise can be obtained in just 9 s using vibration data acquired through real-time monitoring. Therefore, in practical applications, whether to incorporate the feature selection during training can be flexibly determined based on specific scenarios to achieve more accurate prediction results.

**Figure 18:**
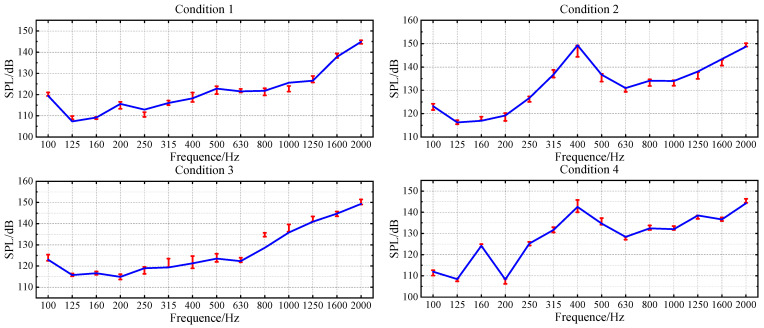
95% confidence interval.

To provide a more comprehensive reference for the reliability of the noise prediction model, this study quantifies prediction uncertainty using the Monte Carlo Dropout (MCDP) method [35]. As an approximate implementation scheme of Bayesian neural networks, MCDP requires no modification to the original training procedure. Instead, it simulates the probabilistic distribution characteristics of model weights simply by keeping the Dropout layers activated during the inference stage. The core principle is as follows: during inference, the Dropout layers randomly deactivate a subset of neurons. By performing *N* independent forward passes on the same input sample, *N* slightly varying predictions are obtained. The distribution formed by these predictions effectively captures the model’s epistemic uncertainty. Building on this, this study adopts a quantile-based method to determine the 95% confidence interval boundaries based on the statistical characteristics of *N* = 400 sampling data. As illustrated in Figure 18, the blue lines represent the true values, and the red lines denote the 95% confidence interval boundaries. The average interval width is 2.50 dB. The confidence intervals are narrow for most frequency bands and encompass the true values. This demonstrates that the ADE-PNN-ResNet model not only achieves high prediction accuracy but also exhibits stable and reliable predictive capability across most frequency ranges.

In the 400 Hz band, the prediction accuracy is significantly improved after introducing feature selection, but the width of the 95% confidence interval is relatively large, with an average of 5.2 dB across four operating conditions. Improved accuracy does not indicate sufficient prediction confidence of the model in this 1/3–octave band. The 400 Hz band likely corresponds to a sensitive region of the hull structure’s dynamic response, where radiated noise is highly sensitive to changes in input vibration features. Feature selection retains only those variables most strongly linked to the physical mechanisms governing radiated noise, enabling the model to focus on the key features and thereby improving prediction accuracy. Nevertheless, precisely because predictions in this band depend critically on a small set of dominant features, if the MCDP randomly masks some neurons during inference and accidentally interrupts the transmission path of these key features, the model struggles to maintain stable output, resulting in high prediction uncertainty. The wider confidence interval for this band is not a model flaw but rather underscores the necessity of feature selection—its high sensitivity to feature quality demands precise screening of core variables to eliminate redundant information interference.

**Figure 19:**
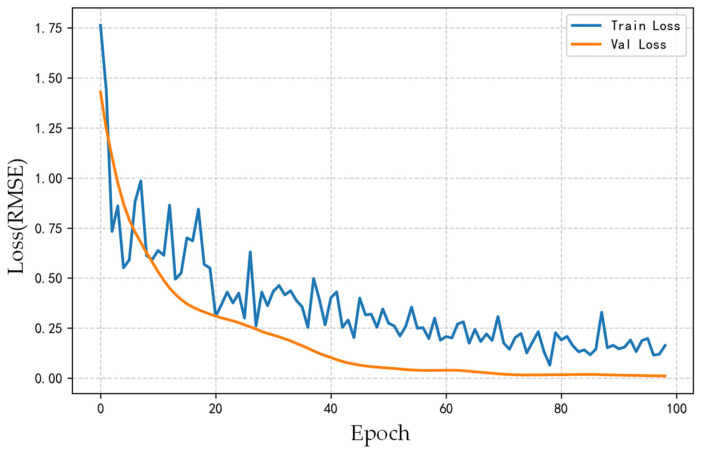
Loss curve of the model for the 1/3-octave band centered at 800 Hz.

Notably, after the introduction of feature selection, the prediction performance at the 800 Hz frequency band under Condition 3 did not significantly improve, with the confidence interval exceeding the true values. However, the prediction errors at 800 Hz for Conditions 1, 2, and 4 remain at low levels. By analyzing the model training process (as shown in Figure 19) and the results from multiple validation conditions, it is observed that the loss function corresponding to the 800 Hz band shows a stable convergence trend over training epochs. The prediction performance for other conditions is satisfactory, indicating that the model has effectively learned the vibration-noise transmission characteristics at this frequency band under typical scenarios, which confirm the good generalization capability of the model across most practical conditions.

However, the prediction result for Condition 3 at 800 Hz shows significant deviation. This suggests that the actual vibration-noise transmission characteristics under Condition 3 differ from the mapping relationships learned by the model. Meanwhile, the training samples fail to fully characterize the vibro-acoustic transfer function of this frequency band under such operating conditions, which ultimately results in the model’s inability to accurately predict the actual noise level of Condition 3 in this frequency band.

### 4.4. Impact of Training Dataset Size on Prediction Accuracy of ADE-PNN-ResNet

**Figure 20:**
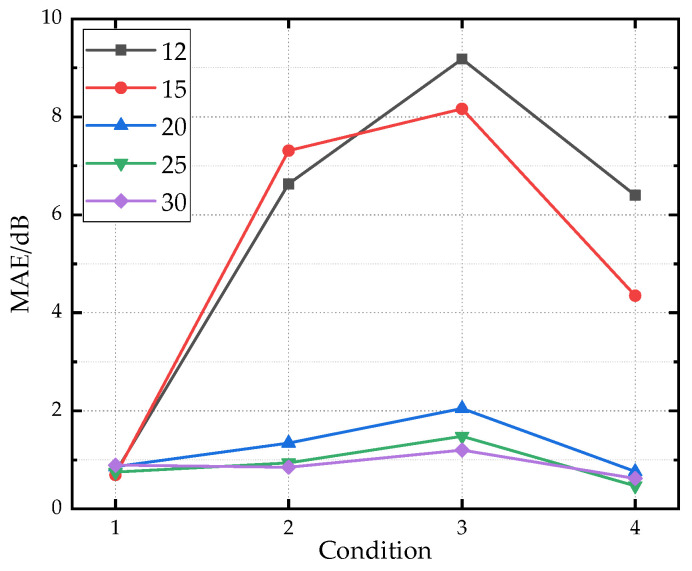
The prediction error under different training sample sizes.

To validate the impact of training sample size on prediction results, random subsets of 12, 15, 20, and 25 samples are selected from the original set of 30 training samples while keeping the validation dataset unchanged. Figure 20 presents line plots of prediction errors for each condition under different training sample sizes. The model performance with 25 samples is similar to that with 30 samples, indicating that the training dataset at this scale is already sufficiently comprehensive to support model learning. When the number of training samples was reduced from 30 to 15 and 12, significant differences in prediction performance are observed across validation conditions. The prediction performance of Condition 1 remains nearly unaffected by the decrease in training sample number, while that of Conditions 2, 3, and 4 drops sharply. The core reason for this phenomenon is that the training dataset still retains the vibration characteristics corresponding to the operating states under Condition 1, whereas those associated with Conditions 2, 3 and 4 are insufficiently represented, directly impairing the model’s generalization ability. Increasing the sample size improves prediction accuracy primarily by better covering a wider range of operating conditions, which allows the neural network to learn the underlying patterns of vibration-to-noise transmission more effectively.

Obtaining full-scale ship sea trial data is costly, making it often difficult to construct large-scale datasets. The small-sample problem has become a key bottleneck restricting the engineering application of the model. To address this, small-sample enhancement strategies can be introduced: data augmentation based on the physical characteristics of vibration and noise signals to expand data distribution; or meta-learning methods to acquire general modeling capabilities from multi-source small-sample tasks, thereby improving the model’s rapid adaptability to new full-scale ship scenarios.

## 5. Conclusions

To meet the engineering requirement for rapid prediction of underwater vehicle radiated noise, this paper proposes the ADE-PNN-ResNet method based on monitoring data-driven modeling. It is established through vibration feature selection and nonlinear vibration-acoustic transfer function fitting, with the main research conclusions summarized as follows:

(1) Traditional numerical methods demand substantial computational resources and are time-consuming, while the OTPA method relies on complex transfer path modeling and comprehensive sensor coverage. This paper replaces traditional physical modeling with a neural network. Addressing the complex nonlinear relationship between underwater structural vibration and radiated noise, the PNN module innovatively integrates RBFNN and MLP in parallel to achieve synergistic complementarity between local details and global trends. Residual connections are further adopted to enhance nonlinear expression capability while avoiding gradient vanishing.
(2) Vibration data from a single frequency band cannot capture the interdependencies among different bands. This paper introduces multi-band vibration feature input, fully considering the synergistic influence of different vibration bands on the target noise. The ADE module is used for joint screening of vibration sensors and frequency bands, extracting features strongly correlated with the target noise bands. This effectively eliminates redundant information and achieves dimensionality reduction, providing high-quality input for subsequent models and enhancing generalization performance.
(3) This study adopts a “single-band model training + full-band integration” modeling strategy. By synergistically combining ADE-based feature selection with the PNN-ResNet architecture, the method achieves high prediction accuracy across the 1/3-octave frequency bands (100–2000 Hz): 96% of the bands exhibit absolute prediction errors below 3 dB. Meanwhile, the average inference time for full-band prediction is only 9 s, meeting the real-time response and accuracy requirements of engineering scenarios.

It should be noted that the proposed method is validated based on scaled model lake test data. However, the real marine environment is subject to complex interferences such as ocean currents and biological noise. Therefore, the anti-interference performance of the model proposed in this paper still needs to be verified and optimized in practical scenarios. In addition, the layout and cost constraints of sensors on actual ships should be considered. Currently, the study models radiated noise as a single output without separating the contributions of different noise sources such as machinery noise and flow noise. In future work, by decoupling the vibration-acoustic transfer paths of various noise sources, the accuracy and applicability of the model in complex acoustic environments will be further improved, providing more comprehensive technical support for the refined prediction and control of radiated noise from underwater vehicles.

## Figures and Tables

**Table 1:** Excitation state.

Exciter	State 1	State 2	State 3	State 4	State 5	State 6
#1	on	-	on	-	-	-
#2	on	on	-	-	-	-
#3	on	on	on	on	on	-
#4	on	on	on	on	-	on

**Table 2:** Validation dataset conditions.

Condition	Model Diving Depth/m	The States of Exciters	Navigational Speed
1	5	state 2	low
2	13	state 5	high
3	5	state 3	low
4	13	state 6	medium

**Table 3:** Comparison of prediction errors among models.

Condition	MAE (dB)					RMSE (dB)				
	PNN-ResNet	PNN	RBFNN	MLP	SVR	PNN-ResNet	PNN	RBFNN	MLP	SVR
1	1.55	1.97	4.59	1.04	2.96	1.73	2.34	5.28	1.43	3.44
2	1.07	1.82	2.44	1.49	2.25	1.35	2.05	3.01	1.87	2.68
3	2.39	2.41	2.23	3.53	1.93	2.97	3.05	2.54	4.55	2.14
4	0.87	1.14	0.43	2.68	1.12	1.14	1.33	0.54	3.58	1.19
Mean	1.47	1.84	2.42	2.19	2.07	1.80	2.19	2.84	2.86	2.36

**Table 4:** Prediction accuracy.

Error	PNN-ResNet	PNN	RBFNN	MLP	SVR
<3 dB	91%	86%	70%	82%	78.6%
<2 dB	70%	64%	55%	60%	59%

**Table 5:** Feature selection results at 400 Hz.

**Vibration Measurement Points**	S3	S4	S5	S6	S7	S8	S9	S11	S18	S20
**1/3-Octave Band Center Frequenc** **y (Hz)**	200			400		630			1250	

## Data Availability

The datasets generated and analyzed during the current study are not publicly accessible as they are part of an ongoing research project with confidentiality agreements. For non-commercial research purposes, qualified researchers may request access to the data by contacting the corresponding author ([heujifang@163.com]) with a detailed research proposal. All requests will be reviewed to ensure compliance with ethical and project-related requirements before data sharing.
